# Trends in Research about COVID-19 Vaccine Documented through Bibliometric and Visualization Analysis

**DOI:** 10.3390/healthcare10101942

**Published:** 2022-10-04

**Authors:** Wei-Ting Wei, Ching-Kuo Wei, Chin-Chu Wu

**Affiliations:** 1 School of Health Care Administration, Taipei Medical University, Taipei 110, Taiwan; 2 MA Curating Cultures, SOAS University of London, London WC1H 0XG, UK; 3 Department of Healthcare Administration, Asia Eastern University of Science and Technology, New Taipei 220, Taiwan; 4 Department of Diagnostic Radiology, Shin Kong Wu Ho-Su Memorial Hospital, Taipei 111, Taiwan

**Keywords:** COVID-19, vaccine, bibliometric analysis, visualization

## Abstract

Due to COVID-19, people’s lives have changed greatly. In accordance with former experience, an efficacious vaccine is the most effective way to curb the pandemic; thus, many researchers have published related publications in the short term. Hence, this study aims at using bibliometric analysis and visualization to document research trends regarding COVID-19 vaccines, and offer some directions and suggestions for future research. Initially, all eligible publications were downloaded from Web of Science on 1 January 2022. Subsequently, some publications published before December 2019 were removed since COVID-19 did not occur before that date. Finally, Microsoft Excel is used for bibliometric analysis to analyze publication date, author, affiliation, country, publication title, publisher, research area, document type, and language, and visualized software (VOSviewer) is used to visualize author, affiliation, country, and keywords. After analyzing a total of 17,392 publications, the results show that the overall research trend was upward. Moreover, the prominent authors, institutions, and countries inclined towards regional cooperation instead of international cooperation. Furthermore, the most popular research areas were immunology and medicine (general and internal). Ultimately, COVID-19, vaccine, and SARS-CoV-2 were the top 3 keywords. In conclusion, this study shows the approximate research trend for COVID-19 vaccine during the completely first two years of the pandemic. The research focuses moved from safety, effectiveness, and immunology at the early stage to the optimal allocation strategies for COVID-19 vaccine, and eventually to public attitudes and acceptance towards COVID-19 vaccination.

## 1. Introduction

COVID-19 (Coronavirus Disease-19), which is precipitated by SARS-CoV-2 (Severe Acute Respiratory Syndrome Coronavirus 2), was first reported in Wuhan at the end of 2019. On 30 January 2020, the World Health Organization (WHO) announced that the disease was a “Public Health Emergency of International Concern, PHEIC” [1]. According to WHO, there have been 545,226,550 confirmed cases of COVID-19 globally, including 6,334,728 deaths from its outbreak to 1 July 2022 [2]. In the light of this detrimental situation, policymakers in each country have carried out a variety of interventions, such as requiring face masks when people go out or take public transportation, support for telecommuting, promoting online studies, restricting travel and even lockdown, so as to decrease the number of people suffering from COVID-19.

According to former experience, the development of vaccines and vaccination programs have led to widespread and important improvements to public health and health care throughout the world [3]. Moreover, Zhai [4] indicated that there is no ultimate weapon to fight against a coronavirus apart from vaccines, and Jeyanathan [5] deemed that the world will not return to its pre-pandemic normalcy until vaccines that are safe and effective become available, and a global vaccination program is conducted successfully. Therefore, it is urgent to have efficacious vaccines to alleviate the dissemination of COVID-19, and control this pandemic.

Bibliometric analysis, which is dubbed bibliometrics as well, enables evaluation of scientific publications through statistical measures, and represents an efficacious method to measure and quantitatively depict their impacts [6]. There are numerous virtues of bibliometric analysis. Firstly, Liao [7] mentioned that bibliometrics allows scholars and researchers to study specific research area by means of analyzing citations, co-citations, geographical distribution and word frequency, and yield useful conclusions. In addition, Zhou [8] pointed out that bibliometric analysis can not only depict the trends and distribution of publications including the impacts and citations but also reflect health policy decisions, the input of medical resources and further social phenomena. Most important of all, it is considered to be helpful for identification of emerging outbreaks of infectious diseases, when bibliometrics can unite novel visualization methods for scientific information [9].

It is evident that COVID-19 vaccine is a crucial issue at this time. Although Ahmad [10], Chen [11], Pratici and Xu [12] have used bibliometric analysis on publications as regards this sphere before, no study has analyzed related publications published before December 2021 to show the research trend during the complete first two year period of the pandemic. Therefore, this study aims at using bibliometric analysis and visualization to organize data on previous research trends, and offer a number of directions and recommendations for future research.

## 2. Materials and Methods

### 2.1. Conceptual Framework and Research Procedure

**Figure 1:**
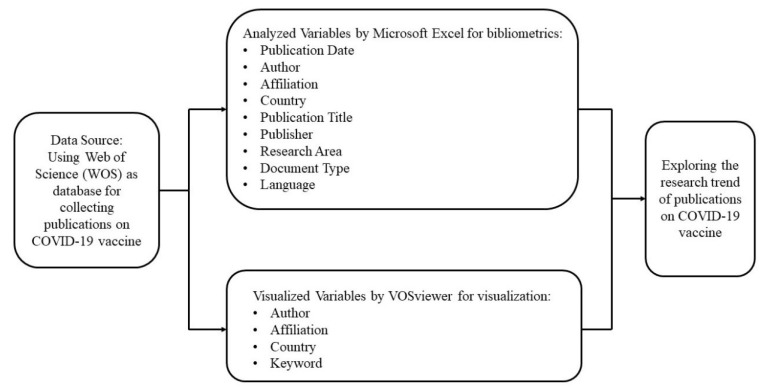
The conceptual framework in this study.

**Figure 2:**
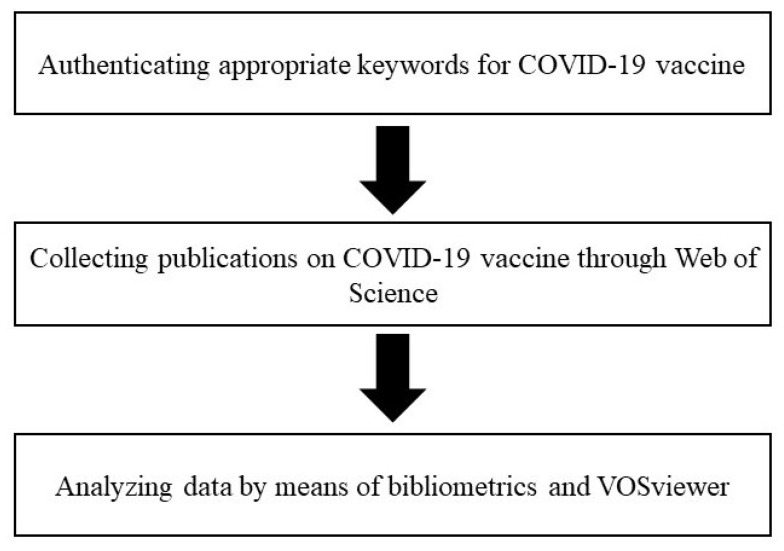
The research procedure in this study.

Based on the research purpose, the initial research procedure was to affirm suitable keywords on COVID-19 vaccine so as to collect appropriate data for analysis. Subsequently, Web of Science (WOS) was used as database for amassing eligible publications. Finally, bibliometric analysis and visualization using VOSviewer were employed for analyzing publications extracted from WOS. The conceptual framework and research procedure in this study are shown in Figure 1 and Figure 2 respectively.

### 2.2. Data Source

It is a prevalent outlook that Science Citation Index Expanded (SCIE) and Social Science Citation Index (SSCI) are the most frequently-used databases when people carry out bibliometric analysis. Web of Science (WOS) is used in this study to amass publications because it contains the most influential and important publications in the scientific world, and is the most frequently utilized database as researchers undertake bibliometric analysis [13].

Apart from the merits mentioned above, there are manifold virtues of WOS. First and foremost, Moura [14] pointed out that WOS has a prominent position as a pioneer in bringing together publications from more than 100 spheres of knowledge. Moreover, WOS is also deemed as one of the most comprehensive databases in several domains of scientific knowledge [15]. Last but not least, WOS comprises publications with higher Impact Factor (IF) in comparison with other databases (e.g., Scopus and Google Scholar) [16]. Hence, WOS is the most ideal database to collect publications for this study.

After choosing Web of Science as the database, corroborating fit keywords for searching is the next step. In this study, the term “coronavirus disease 2019” OR “COVID-19” OR “severe acute respiratory syndrome coronavirus 2” OR “2019-nCov” OR “SARS-CoV-2” (Topic) and vaccin* (Topic) were used as keywords to search publications on COVID-19 vaccine, and a total of 17,393 publications were collected. All publications were downloaded on the same day (1 January 2022) in order to prevent bias occurring in view of the update of the WOS on a daily basis.

### 2.3. The Principle of Selecting Core Publications

**Figure 3:**
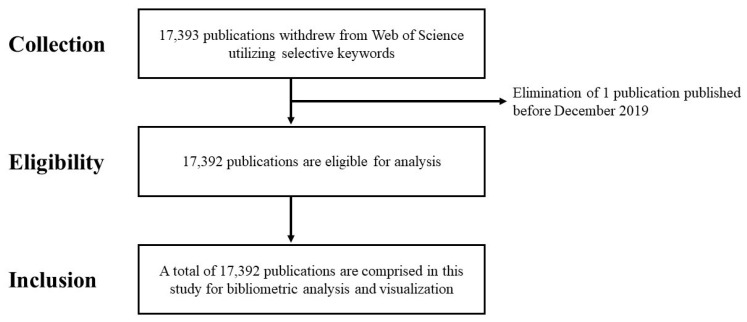
The flowchart of selecting core publications on COVID-19 vaccine.

In this study, all publications downloaded from WOS, irrespective of their research areas, were incorporated since they reflect different dimensions in respect of COVID-19 vaccine. However, the outbreak of COVID-19 occurred in December 2019, and therefore one publications was removed as it was published before that date. Finally, a total of 17,392 publications were included in this study. The selection process is demonstrated in Figure 3.

### 2.4. Data Analysis

In this study, Microsoft Excel 2016 was utilized for bibliometric analysis, and VOSviewer version 1.6.17 was employed for visualization. Since it is recognized as an effective way to assess scientific progress when bibliometrics is combined with visualized mapping [17], VOSviewer, which was developed by Nees Jan van Eck and Ludo Waltman [18], was taken into consideration. In VOSviewer, the network visualization reveals concepts on the basis of their importance. Generally speaking, the larger circle size or font dimension implies greater productivity or citations. Furthermore, the color of the circle illustrates that which term belongs to which cluster. Generally, items with identical color hint that they belong to the same cluster [19]. For instance, the relationship between keywords is decided based on the number of articles where the keywords occur together [20], and the size of the node for each keyword in the map symbolizes its frequency of occurrence [21]. Therefore, Microsoft Excel was used to analyze for publication date, author, affiliation, country, publication title, publisher, research area, document type, and language, and VOSviewer was used to visualize author, affiliation, country, and keyword.

## 3. Results

In this section, the 10 subsections summarize data on publication date, author, affiliation, country, publication title, publisher, research area, keyword, document type, and language respectively.

### 3.1. Publication Date

**Figure 4:**
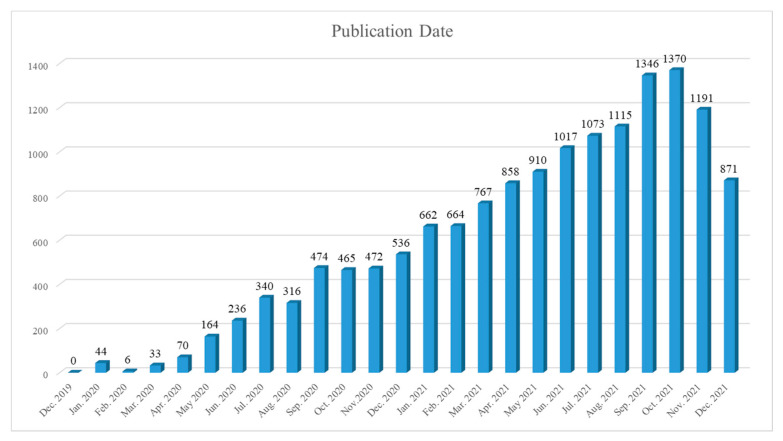
The overall trend of publications on COVID-19 vaccine from December 2019 to December 2021.

Figure 4 shows the overall trend of publications on COVID-19 vaccine from December 2019 to December 2021. By and large, the overall research tendency was upward.

In more detail, the number of publications increased steadily from 0 in December 2019 to 1346 in September 2021, and subsequently to 1370 in October 2021. This was the highest point during the timespan.

### 3.2. Author

80,656 authors published their research as regards COVID-19 vaccines. Table 1 shows the top 10 authors. Among them, the most productive author was Mahase E (*n* = 78). The top 2nd to 5th authors were Kumar S (*n* = 61), Dhama K (*n* = 53), Kumar A (*n* = 53) and Zhang Y (*n* = 51).

**Figure 5:**
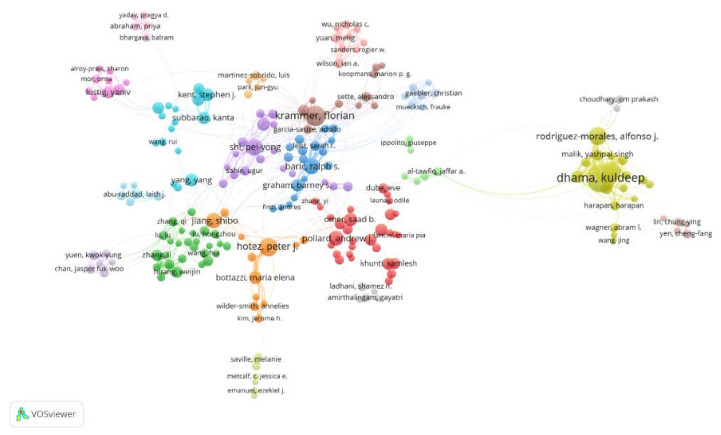
The cooperation chart for the authors, using network visualization through VOSviewer.

Furthermore, Figure 5 shows the collaborations of the authors through network visualization using VOSviewer. Although there are 20 clusters in total in the figure, only 6 clusters are mentioned here. Among them, the authors in the orange (e.g., Hotez Peter J.) and blue (e.g., Baric Ralph S.) clusters had close collaborations with those in the red cluster (e.g., Pollard Andrew J.). The authors in the green cluster (e.g., Huang Weijin) inclined to cooperate with the authors in the orange cluster, and the authors in the purple (e.g., Shi Pei-Yong) cluster tended to collaborate with the authors in the blue cluster. The authors in the khaki cluster (e.g., Dhama Kuldeep) did not have cooperation with the mainstream.

To sum up, the results illustrated that copious authors made contributions on this field, and the prominent authors did not have too many interchanges.

### 3.3. Affiliation

In this study, downloaded papers showed author affiliations to 15,696 institutions supporting research regarding COVID-19 vaccine. Table 2 demonstrates the top 10 organizations. Among them, the top 2 organizations were Harvard University (*n* = 597) and University of London (*n* = 580). The third most productive institution was the University of California System (*n* = 457).

**Figure 6:**
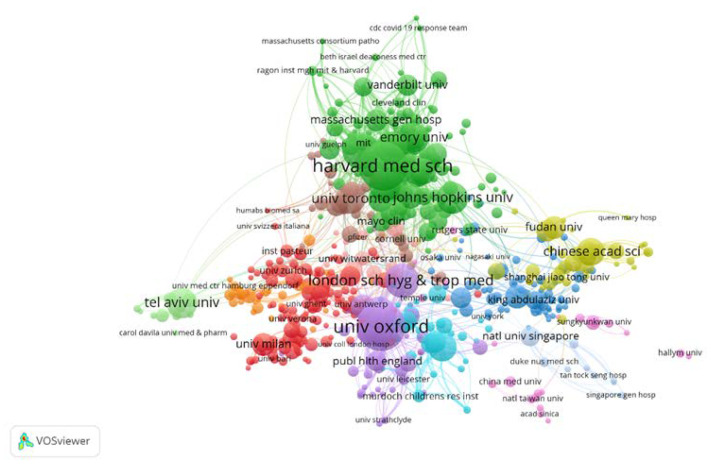
The cooperation chart for institutional affiliations, using network visualization through VOSviewer.

Moreover, Figure 6 shows the collaborations of the organizations through network visualization using VOSviewer. Even though there are 13 clusters in the figure, only 7 clusters are mentioned here. Among them, the institutions in the green cluster (e.g., Harvard Medical School) were mostly located in United States, and the organizations in the brown cluster (e.g., University of Toronto) were chiefly situated in Canada. There were close alignments between the institutions in these two clusters. Furthermore, the organizations in the red cluster (e.g., University of Milan) were principally sited in Europe, and the organizations in the purple cluster (e.g., University of Oxford) were primarily sited in United Kingdom. Aside from mutual alignments, the institutions in these two clusters inclined to collaborate with those in United States and Canada. Moreover, the affiliations in the blue cluster (e.g., King Saud University) were mainly located in the Arab countries, and the organizations in the khaki cluster (e.g., Chinese Academy of Sciences) were largely situated in China. Apart from reciprocal alignments, the institutions in these last-mentioned two clusters also mostly cooperated with those in United States and United Kingdom instead of those in Canada and Europe. In addition, the institutions in the fluorescent green cluster (e.g., Tel Aviv University) were more likely to carry out research independently, albeit they had inclinations to collaborate with those in United States and Canada. In short, the top 10 institutions were largely located in United States and United Kingdom, and there was a tendency towards regional alignments rather than international collaborations.

### 3.4. Country

**Figure 7:**
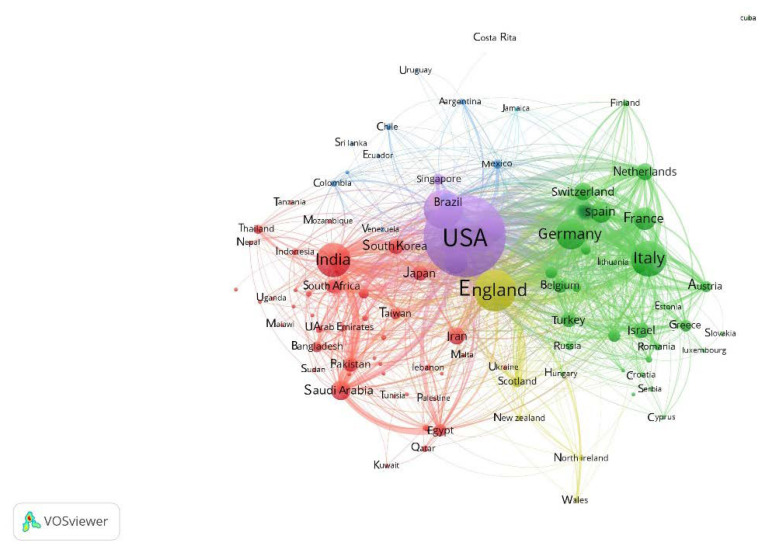
The cooperation chart of the countries for journals publishing research papers on COVID-19, using network visualization through VOSviewer.

The analysis showed a total of 178 countries contributing to this research relating to COVID-19 vaccine. Table 3 demonstrates the top 10 countries. Among them, the United States (*n* = 5640; 21.388%) was the most prolific nation, and the United Kingdom (*n* = 2181; 8.271%) and China (*n* = 1650; 6.257%) were the second and third most productive countries, respectively. The top 4 to 6 countries were Italy (*n* = 1403; 5.320%), India (*n* = 1326; 5.028%) and Germany (*n* = 1078; 4.088%) in order. These six countries contributed nearly 50% of publications. It was found that: America (the U.S.A. and Canada) ranked first at 24.376% (21.388% in the U.S.A.), followed by Europe (including the U.K., Italy, Germany, France, and Spain) at 22.453%, while in the third place was Asia (China and India) at 11.255%, and finally Australia (2.746%). Figure 7 shows the collaborations of the countries through network visualization using VOSviewer. There are 6 clusters all told in the figure, of which 5 clusters are mentioned here. Among them, the countries in the purple (e.g., United States, China, Canada and Australia) and yellow (e.g., United Kingdom) clusters were core in this field. The countries in the green cluster (e.g., Italy) were mainly situated in Europe, and the countries in the red cluster (e.g., India) were chiefly located in Asia. The countries in these two clusters tended to collaborate with those in the purple and yellow clusters, but were less likely to cooperate mutually. The nations in the blue cluster (e.g., Mexico) were primarily sited in Latin America, and they inclined to collaborate with the countries in the purple, red and green clusters rather than those in the yellow cluster.

### 3.5. Publication Journal Title

In this study, 2736 journals were found to have published research papers dedicated to COVID-19 vaccines. Table 4 shows the top 10 journals. Among them, Vaccines (*n* = 630; 3.618%) was the most productive journal. The top 2nd and 3rd journals were BMJ British Medical Journal (*n* = 379; 2.176%) and Vaccine (*n* = 310; 1.780%), respectively. In summary, these productive journals chiefly specialized in medicine. 

### 3.6. Publisher

The study identified 421 publishers which published research referring to COVID-19 vac cine. Table 5 demonstrates the top 10 publishers. Among them, the top 4 publishers were Elsevier (*n* = 3738; 21.491%), Wiley (*n* = 1773; 10.194%), Springer Nature (*n* = 1762; 10.130%) and MDPI (*n* = 1733; 9.964%) respectively. The top 4 publishers released slightly more than 50% of publications.

### 3.7. Research Area

In this study, 135 research areas were identified. The top 10 research fields are reflected in Table 6. Among them, the top 2 research fields were immunology (*n* = 2721; 10.567%) and general and internal medicine (*n* = 2268; 8.808%). The third most popular research area was research on experimental medicine (*n* = 1746; 6.781%). Briefly, the top 3 research spheres accounted for marginally more than 25% of publications.

### 3.8. Keywords

25,176 keywords all told were used by research teams while publishing their research results. Table 7 reveals the top 20 keywords. Among them, the most used keywords were COVID-19 (*n* = 9693) and vaccine (*n* = 9240). The top 3 to 5 keywords were SARS-CoV-2 (*n* = 7204), Cov (*n* = 6328) and coronavirus (*n* = 4222), all relating to the virus triggering the COVID-19 pandemic.

**Figure 8:**
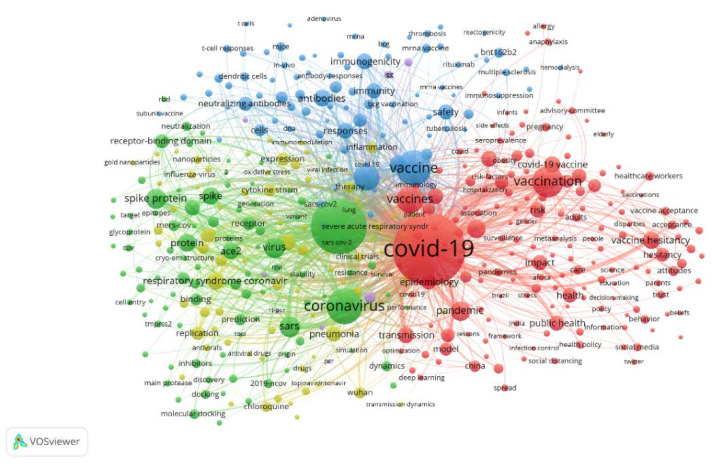
The main keywords of publications on the policy of COVID-19 vaccination through network visualization using VOSviewer.

Figure 8 reveals the interrelationships of the focal keywords through network visualization using VOSviewer. Although there are 5 clusters in all in the figure, only 4 clusters are described here. Among them, the keywords in the red cluster (e.g., COVID-19, COVID-19 vaccine, vaccine hesitancy and public health) used comprehensive edges to explore COVID-19 vaccine. The keywords in the blue cluster (e.g., antibodies, immunity and immunogenicity) principally implicated immunology relating to COVID-19 vaccine, and the keywords in the green cluster (e.g., SARS-CoV-2, coronavirus and virus) mostly involved virology. The keywords in the yellow cluster (e.g., pneumonia and replication) described other facets. For these four clusters, the keywords in the red, blue and green clusters had close links reciprocally. However, the keywords in the yellow cluster had close association with those in the green cluster, but their connections with the keywords in the red and blue clusters were limited.

**Figure 9:**
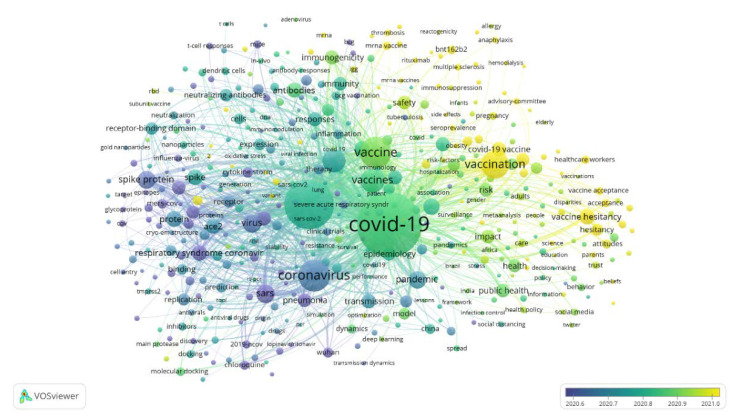
The major keywords of publications on the policy of COVID-19 vaccination through overlay visualization using VOSviewer.

Figure 9 displays the evolution of the keywords of publications on COVID-19 vaccine through overlay visualization using VOSviewer. The research focuses varied from those shown in the purple (e.g., pneumonia and Wuhan) and navy blue (e.g., coronavirus and spike protein) colors to those revealed in the blue-green (e.g., SARS-CoV-2) and bright green (e.g., COVID-19 and vaccines) colors, and finally to those reflected in the fluorescent green (e.g., public health and health) and yellow (e.g., vaccine hesitancy) colors.

### 3.9. Document Type

The study included 18 document types in all. Table 8 demonstrates the top 10 document types. Among them, the main document types was articles (*n* = 9691; 53.212%), while review articles (*n* = 3088; 16.956%) and editorial material (*n* = 1931; 10.603%) ranked as the top 2 and 3, respectively. These three document types made up roughly 80 % of publications.

### 3.10. Language

The stuy found publications in a total of 15 languages. Table 9 shows the top 10 languages. Among them, nearly all publications (*n* = 17,093; 98.275%) were written in English.

## 4. Discussion

### Language Discussion on the Research Trend of Publications on COVID-19 Vaccine

From a macro point of view, the overall research tendency in connection with COVID-19 vaccine was upward. It jibed with the prognoses made by Chen [11] and Noruzi [22]. Hence, we deduce that COVID-19 vaccine was seen as the key to controlling the catastrophe caused by this epidemic [23], so that copious relevant publications were published in a short term.

Global collaborations between primary authors, affiliations and countries were not as frequent as regional alignments, perhaps because researchers and governments were desperate to find effective vaccines so as to put the brakes on the COVID-19 pandemic. Many related publications were issued in a short time, and collaborations may have reflected regional linkages. Remarkably, there were altogether 169 vaccines in clinical development as well as 198 vaccines in pre-clinical development on 8 July 2022 [24], showing widespread commitment to action. The top 10 institutions identified in the study were principally sited in United States, the most productive country, and United Kingdom, the second most prolific country.

Via bibliometric analysis of publication titles, publishers, research areas, and keywords, we observe that the research spheres that the top 10 journals reflected were largely associated with immunology and medicine, which accounted for approximately 25% of publications. These two research fields were also the top 3 research areas. Therefore, the results were consistent. The percentages that the top 10 publication titles constituted among the top 10 publishers was not high, but the top 4 publishers made up roughly 50% of publications. We reason that the reason for this apparently contradictory result was that several journals published by the top 10 publishers had made contributions rather than the fact that these publishers focused on only one journal publishing relevant publications. Moreover, the research fields, which prime keywords are implicated in, were wide-ranging. The inference is that the percentage representation of each research area was not high. However, the main keywords were related to the top 10 research areas; thus, the results were congruous.

Lopes [25] asserted that English was the most used language all over the world. In this study, English was the most commonly used language when research teams published their research results, supporting Lopes’ opinion.

## 5. Conclusions and Recommendations

This study demonstrates the approximated research trend for COVID-19 vaccines during the complete first two year period of the COVID-19 pandemic. Since COVID-19 is a new epidemic, the research for COVID-19 vaccine initially concentrated on safety, effectiveness and immunology. After vaccine development, it was certain that the demand for COVID-19 vaccine would exceed its supply. Therefore, the optimum allocation strategy for COVID-19 vaccine became a research focus in order to maximize the benefits. Finally, researchers inclined to zero in on public attitudes and acceptance towards COVID-19 vaccination after the supply of COVID-19 vaccine outstripped the demand. Hence, this study offers some reference for future research, so that future researchers can foresee possible trends if a new pandemic breaks out again in the future.

As of 9 July 2022, COVID-19 and COVID-19 vaccination are still ongoing. Therefore, researchers must continue undertaking relevant research, meaning that more and more publications will be published in the future. For those concerned with COVID-19 vaccines, it is predicted that the nub of the research will focus on the effectiveness of confrontation with different virus variants as well as people’s attitudes towards continuous COVID-19 vaccination.

Pierce [26] pointed out that research articles in core publication titles became increasingly similar in their bibliometric features as the disciplines mature. In this study, we find that the percentage of the document type “article” among the publications on COVID-19 vaccine was about 53.2%, and thereby we infer that this sphere was still immature at the end of 2021. Hence, we suggest that future researchers should continue undertaking research in connection with COVID-19 vaccine. Moreover, if a future research objective is to develop a knowledge graph for COVID-19 vaccine literature, the deep neural networks (DNN) method can be adopted to achieve the research purpose. 

This study has several limitations. First of all, different researchers may use different words to express the same thing, and thereby some publications with varied expressions may be excluded while collecting publications from Web of Science. Furthermore, Web of Science is used as database for amassing publications in this study, and some publications only included in other databases (e.g., Scopus, PubMed and Google Scholar) may be excluded. Moreover, this study only gathers publications released before 31 December 2021; thus, some newer publications must have been missed. Thus, updated information on research trends will require future research, since the problem of COVID-19 will not be thoroughly sorted out for some years to come.

## Figures and Tables

**Table 1:** The top 10 authors of publications on COVID-19 vaccines.

Rank	Author	Frequency
1	Mahase E	78
2	Kumar S	61
3	Dhama K	53
3	Kumar A	53
5	Zhang Y	51
6	Krammer F	47
6	Wang J	47
8	Li Y	45
9	Li J	44
10	Liu Y	43

**Table 2:** The top 10 affiliations of publications on COVID-19 vaccines.

Rank	Affiliation and Its Located Country	Frequency
1	Harvard University in United States	597
2	University of London in United Kingdom	580
3	University of California System in United States	457
4	Harvard Medical School in United States	360
5	University of Oxford in United Kingdom	350
6	Johns Hopkins University in United States	307
7	University of Texas System in United States	268
8	Institut national de la santé et de la recherche médicale Inserm in France	263
9	National Institutes of Health (NIH) in United States	252
10	Imperial College London in United Kingdom	249

**Table 3:** The top 10 countries of journals with publications on COVID-19 vaccines.

Rank	Country	Frequency	Percentage
1	United States	5640	21.388%
2	United Kingdom	2181	8.271%
3	China	1650	6.257%
4	Italy	1403	5.320%
5	India	1326	5.028%
6	Germany	1078	4.088%
7	Canada	788	2.988%
8	Australia	724	2.746%
9	France	668	2.533%
10	Spain	591	2.241%

**Table 4:** The top 10 journal titles for publications on COVID-19 vaccines.

Rank	Publication Title (Impact Factor in 2020) and Publisher	Journal Citation Reports (JCR) Category (Category Rank in 2020; Category Quartile in 2020)	Frequency	Percentage
1	Vaccines (4.422) published by MDPI	Immunology in SCIE edition (75/162; Q2) Medicine, Research & Experimental in SCIE edition (63/140; Q2)	630	3.618%
2	BMJ British Medical Journal (39.89) published by BMJ Publishing Group	Medicine, General & Internal in SCIE edition (5/167; Q1)	379	2.176%
3	Vaccine (3.641) published by Elsevier	Immunology in SCIE edition (99/162; Q3) Medicine, Research & Experimental in SCIE edition (76/140; Q3)	310	1.780%
4	Human Vaccines & Immunotherapeutics (3.452) published by Taylor & Francis	Biotechnology & Applied Microbiology in SCIE edition (68/159; Q2) Immunology in SCIE edition (106/162; Q3)	282	1.619%
5	Frontiers in Immunology (7.561) published by Frontiers Media SA	Immunology in SCIE edition (24/162; Q1)	245	1.407%
6	International Journal of Environmental Research and Public Health (3.39) published by MDPI	Environmental Sciences in SCIE edition (118/274; Q2) Public Environmental & Occupational Health in SCIE edition (68/203; Q2) Public Environmental & Occupational Health in SSCI edition (42/176; Q1)	204	1.171%
7	The New England Journal of Medicine (91.253) published by Massachusetts Medical Society	Medicine, General & Internal in SCIE edition (1/167; Q1)	203	1.166%
8	PLOS ONE (3.24) published by Public Library of Science (PLOS)	Multidisciplinary Sciences in SCIE edition (26/72; Q2)	196	1.126%
9	Nature (49.962) published by Nature Research	Multidisciplinary Sciences in SCIE edition (1/72; Q1)	171	0.982%
10	Viruses-Basel (5.048) published by MDPI	Virology in SCIE edition (10/37; Q2)	167	0.959%

**Table 5:** The top 10 publishers of journals with papers on COVID-19 vaccines.

Rank	Publisher	Frequency	Percentage
1	Elsevier	3738	21.491%
2	Wiley	1773	10.194%
3	Springer Nature	1762	10.130%
4	MDPI	1733	9.964%
5	Taylor & Francis	931	5.353%
6	Frontiers Media SA	767	4.410%
7	BMJ Publishing Group	735	4.226%
8	Nature Portfolio	600	3.450%
9	Oxford University Press	378	2.173%
10	SAGE	345	1.984%

**Table 6:** The top 10 research areas of publications on COVID-19 vaccines.

Rank	Research Area	Frequency	Percentage
1	Immunology	2721	10.567%
2	General and Internal Medicine	2268	8.808%
3	Research on Experimental Medicine	1746	6.781%
4	Public Environmental and Occupational Health	1518	5.895%
5	Science Technology and other Topics	1303	5.060%
6	Pharmacology and Pharmacy	1180	4.583%
7	Infectious Diseases	1146	4.451%
8	Biochemistry and Molecular Biology	1143	4.439%
9	Microbiology	764	2.967%
10	Virology	697	2.707%

**Table 7:** The top 20 keywords of publications on COVID-19 vaccines.

Rank	Keyword	Frequency
1	COVID-19	9693
2	Vaccine	9240
3	SARS-CoV-2	7204
4	Cov	6328
5	Coronavirus	4222
6	Immune	2587
7	Infect	2091
8	Virus	2020
9	Health	1969
10	Antibody	1960
11	Disease	1871
12	Protein	1862
13	Cell	1797
14	Syndrome	1684
15	Respiratory	1537
16	Response	1531
17	Influenza	1356
18	Spike	1327
19	Model	1148
20	Receptor	1017

**Table 8:** The top 10 document types of publications on COVID-19 vaccine.

Rank	Document Type	Frequency	Percentage
1	Article	9691	53.212%
2	Review Article	3088	16.956%
3	Editorial Material	1931	10.603%
4	Letter	1605	8.813%
5	Early Access	786	4.316%
6	News Item	502	2.756%
7	Meeting Abstract	465	2.553%
8	Correction	95	0.522%
9	Book Chapter	21	0.115%
10	Proceedings Paper	7	0.038%

**Table 9:** The top 10 used languages of publications on COVID-19 vaccines.

Rank	Language	Frequency	Percentage
1	English	17,093	98.275%
2	German	130	0.747%
3	Spanish	66	0.379%
4	French	56	0.322%
5	Portuguese	15	0.086%
6	Polish	7	0.040%
7	Hungarian	6	0.034%
7	Russian	6	0.034%
9	Turkish	4	0.023%
10	Icelandic	3	0.017%
